# Cross-cultural adaptation of the Perceived Risk of HIV Scale in Brazilian Portuguese

**DOI:** 10.1186/s12955-021-01760-6

**Published:** 2021-04-09

**Authors:** Thiago S. Torres, Paula M. Luz, Luana M. S. Marins, Daniel R. B. Bezerra, Celline C. Almeida-Brasil, Valdilea G. Veloso, Beatriz Grinsztejn, Daphna Harel, Brett D. Thombs

**Affiliations:** 1 Instituto Nacional de Infectologia Evandro Chagas, Fundação Oswaldo Cruz, Av. Brasil 4365, Manguinhos, Rio de Janeiro, 21040-900 Brazil; 2 Research Institute of the McGill University Health Centre, Montreal, Canada; 3 Department of Applied Statistics, Social Science, and Humanities, New York University, New York, NY USA; 4 Center for Practice and Research and the Intersection of Information, Society, and Methodology, New York University, New York, NY USA; 5 Lady Davis Institute for Medical Research, Jewish General Hospital, Montréal, Québec Canada; 6 Departments of Psychiatry; Epidemiology, Biostatistics and Occupational Health; Medicine; Psychology; Educational and Counselling Psychology, and Biomedical Ethics Unit, McGill University, Montréal, Québec Canada

**Keywords:** Perceived risk of HIV Scale, Psychometric properties, GBM, Transgender, HIV perceived risk, HIV risk-behavior, Brazil

## Abstract

**Background:**

Valid and reliable instruments are needed to measure the multiple dimensions of perceived risk. The Perceived Risk of HIV Scale is an 8-item measure that assesses how people think and feel about their risk of infection. We set out to perform a cross-cultural adaptation of the scale to Brazilian Portuguese among key populations (gay, bisexual and other men who have sex with men and transgender/non-binary) and other populations (cisgender heterosexual men and cisgender women).

**Methods:**

Methodological study with cross-sectional design conducted online during October/2019 (key populations [sample 1] and other populations) and February–March/2020 (key populations not on pre-exposure prophylaxis [sample 2]). Cross-cultural adaptation of the Perceived Risk of HIV Scale followed Beaton et al. 2000 guidelines and included confirmatory factor analysis, differential item functioning (DIF) using the Multiple-Indicator Multiple-Cause model, and concurrent validity to verify if younger individuals, those ever testing for HIV, and engaging in high-risk behaviors had higher scores on the scale.

**Results:**

4342 participants from key populations (sample 1 = 235; sample 2 = 4107) and 155 participants from other populations completed the measure. We confirmed the single-factor structure of the original measure (fit indices for sample 1 plus other populations: CFI = 0.98, TLI = 0.98, RMSEA = 0.07; sample 2 plus other populations: CFI = 0.97, TLI = 0.95, RMSEA = 0.09). For the comparisons between key populations and other populations, three items (item 2: “I worry about getting infected with HIV”, item 4: “I am sure I will not get infected with HIV”, and item 8: “Getting HIV is something I have”) exhibited statistically significant DIF. Items 2 and 8 were endorsed at higher levels by key populations and item 4 by other populations. However, the effect of DIF on overall scores was negligible (0.10 and 0.02 standard deviations for the models with other populations plus sample 1 and 2, respectively). Those ever testing for HIV scored higher than those who never tested (*p* < .001); among key populations, those engaging in high-risk behaviors scored higher than those reporting low-risk.

**Conclusion:**

The Perceived Risk of HIV Scale can be used among key populations and other populations from Brazil.

**Supplementary Information:**

The online version contains supplementary material available at 10.1186/s12955-021-01760-6.

## Background

By 2018, more than 900,000 individuals were living with HIV in Brazil [1]. National Surveillance data indicates that more than 300,000 new HIV infections were diagnosed from 2007 to 2019, 69% of them among males [2]. Like other Latin American countries, the HIV epidemic in Brazil disproportionally affects key populations, with gay, bisexual and other cisgender men who have sex with men (GBM) and transgender women who have sex with men (TGW) facing the highest burden [3]. HIV prevalence is estimated at 18% among GBM [4] and 31% among TGW [5], while it is below 0.5% among the general population [2].

Prior studies have shown that Brazilian GBM and TGW engage to a higher degree in behaviors associated with increased HIV risk, such as condomless receptive anal sex [5–9] and use of alcohol or illicit drugs before or during sex (‘chemsex’) [10]. Although currently the effect of these behaviors may be mitigated by pre-exposure prophylaxis (PrEP) and treatment as prevention, which are available free of charge through the Brazilian Public Health System [11], results from web-based surveys have shown that almost 30% of Brazilian GBM have never heard of PrEP [7] and 17% were never tested for HIV, with higher proportions in lower income regions [12]. In this sense, it is important to identify predictors of engagement in high-risk behavior among key populations in Brazil.

According to the Health Belief Model, individuals who perceive themselves at higher risk of a particular health problem are more likely to engage in behaviors to help reduce their risk [13]. Other theoretical models also consider perceived risk as an important correlate of risk behavior, including the AIDS Risk Reduction Model [14]. Prior studies that have accessed perceived HIV risk among Brazilian GBM and TGW have done so using a single question focusing mainly on the likelihood or the cognitive assessment of risk: “In your opinion, what is your risk of getting HIV in the next year?” with possible response options “No risk”, “Low risk”, “High risk”, “Certain” and “I don’t know/prefer not to answer” [6, 7, 9, 15–17]. However, HIV risk perception is complex, and requires a valid and reliable instrument to measure its multiple dimensions.

The Perceived Risk of HIV Scale [18] is a low burden, self-report 8-item measure developed to assess how people think and feel about their risk of HIV infection based on their previous sexual behavior and covering several dimensions of perceived HIV risk. The scale was initially evaluated using item response theory in a sample of participants recruited from HIV testing and prevention services in Long Beach, California. It assesses three different aspects of perceived HIV risk: cognitive (3-items, e.g., “I think my chances of getting infected with HIV are:”, response options: zero, almost zero, small, moderate, large, very large), intuitive (3-items, e.g., “I feel vulnerable to HIV infection”, response options: strongly disagree to strongly agree), and salience (2-items, e.g., “Getting HIV is something I have:”, response options: never thought about, rarely thought about, thought about some of the time, thought about often). Response format varies from four to six options depending on the item. The total summed score ranges from 10 to 40, calculated by adding the score of each item. Higher scores indicate a higher risk perception. Prior research shows that scores on this measure are correlated with past engagement in high-risk behavior, including number of sex partners, condomless anal sex, and sex under influence of substances [18].

The Perceived Risk of HIV Scale was previously validated in European Portuguese in a sample of the general population and among HIV-uninfected partners of serodifferent couples (mostly heterosexuals). The correlation of the scale’s scores with engagement in high-risk sexual behavior and with HIV testing provided evidence for criterion validity [19]. However, the application of the European Portuguese version of the scale in Brazil may not be appropriate due to significant cultural and language differences between Brazilian and European Portuguese. Moreover, given the disproportionate impact of the Brazilian HIV epidemic among key populations, a specific evaluation of the scale’s validity among these populations is warranted.

This study performed a cross-cultural adaptation of the Perceived Risk of HIV Scale in Brazilian Portuguese among key populations (GBM and transgender/non-binary) and other populations (cisgender heterosexual men and cisgender women) using established guidelines [20]. We then evaluated its reliability (internal consistency) and validity. Furthermore, we tested for differential item functioning (DIF) to assess measurement invariance among the populations included in our study.

## Methods

### Study design

This is a methodological study with cross-sectional design conducted online during October/2019 (key populations [sample 1] and other populations) and February–March/2020 (key populations not on pre-exposure prophylaxis [sample 2]). Cross-cultural adaptation of the Perceived Risk of HIV Scale followed guidelines proposed by Beaton et al. [20]. This study was not registered in any database.

### Step 1: Translation

We have obtained the necessary permissions from the authors of the Perceived Risk of HIV Scale to modify it. Translation of the items of the scale into Brazilian Portuguese was performed by three independent translators (two researchers and a linguistics professor fluent in both languages), after which a meeting was held to discuss and reach a consensus translated version of the scale. Then, three additional independent reviewers (two language teachers and one professional translator) translated the Portuguese version back to English, after which another meeting was held with the six members of the translation team and a mediator who was a member of the research team to compare the original items with the back-translated items and identify where items or words seemed to differ. In this final meeting the team reached an agreed-on version based on the comments, the original items, and the translated items. Next, two experts evaluated the translated items vis-à-vis the original subscales to judge if, in their opinion, they captured the concepts as defined. Finally, a qualitative pretesting of the resulting items was conducted with a small convenience sample to ensure item comprehensibility before moving into the second step of this study. For this, we invited via WhatsApp groups, GBM aged 18 years or older who had previously sought HIV testing and prevention services at Instituto Nacional de Infectologia Evandro Chagas (INI-Fiocruz), Rio de Janeiro, Brazil. An electronic version of the scale was provided and participants were requested to judge the clarity of each item on a scale from 0 to 10, and, if an item was scored as 7 or lower, an additional open text field was provided and the participant was asked to state what was unclear and to provide suggestions to improve clarity. A group meeting of the research team was held to discuss the suggestions made and items were adjusted as needed to improve understanding.

### Step 2: Reliability and construct validity

**Fig. 1:**
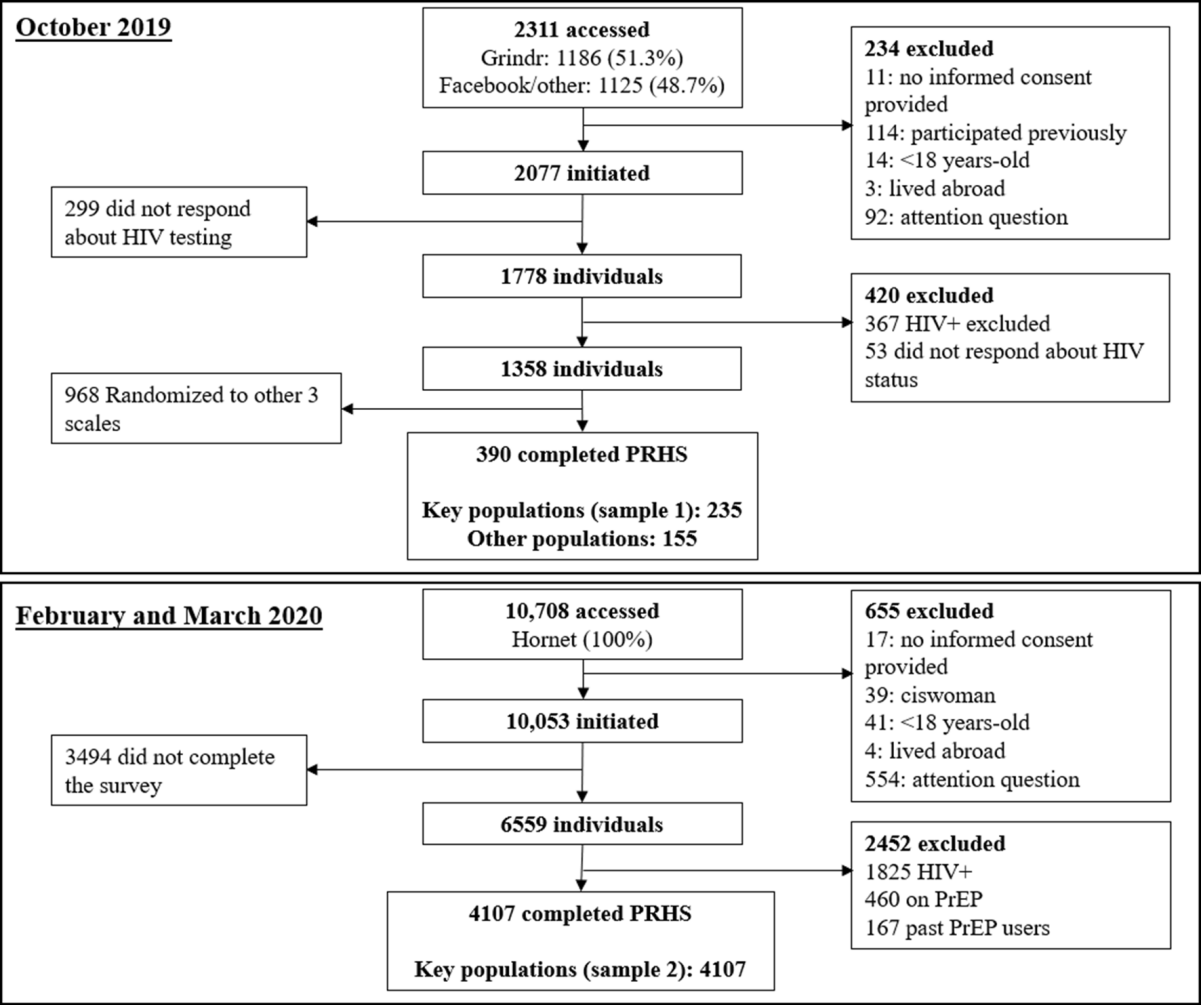
Participant flow chart

A first convenience sample of adult Brazilians was recruited through advertisements on different platforms: (1) Facebook and WhatsApp, two social media apps; and (2) Grindr, a geospatial network app for sexual encounters for GBM and transgender/non-binary people. Participant eligibility included age ≥ 18 years and residency in Brazil. To decrease participant burden, random allocation of eligible participants to different instruments was performed such that each participant only responded to one instrument. Exclusion criteria for this analysis were self-report of HIV positive status, no response in questions about HIV testing or HIV status, an incorrect response to any of three attention questions which were included throughout the survey instrument at approximately every 15 items [21], and having responded to the survey previously (see Fig. 1: Participant flow chart). In order to provide more evidence of the scale’s construct validity, we recruited a second convenience sample including only key populations. This time, the survey was launched through advertisements on Hornet, another geospatial network app for GBM and transgender/non-binary people. The same inclusion and exclusion criteria detailed above were used. Additionally, we excluded from this analysis those who ever used PrEP given that PrEP use reduces the risk of HIV transmission irrespective of one’s sexual behavior [22, 23].

The surveys were programmed on Alchemer®. The researchers checked the usability and technical functionality of the electronic questionnaire in multiple platforms (devices and operating systems) before starting the survey. The survey was open (not password-protected) and not mandatory for app users. Mobile version of the survey had one question per page, and number of pages depended on the chosen answers (adaptative questioning). All survey items had a non-response option (“I don’t want to answer”).

The survey instrument was divided into three sections as follows. Section 1 included items on socio-demographic information (age, race/ethnicity, gender, sexual orientation, education, income, and state of residence), and Sect. 2 included items referring to prior HIV testing and HIV test result. If HIV negative or unknown status, participants answered Sect. 3 which included the items of the translated scale (Additional file 1). As in the original scale [18], response options for the translated scale were on 4, 5 or 6-point Likert type and the total score was calculated by adding the score of each item (with item 4 being reverse coded and some response options for items 1, 2, 5 and 6 recoded). Higher scores indicated greater risk perception. A fourth section was included in the survey launched in Hornet in 2020 with questions about sexual behavior during the previous 6 months (number of male sexual partners, condomless receptive anal sex, sex with HIV positive partner, number of insertive anal intercourses with HIV positive partner), and use of stimulants. These questions (in addition to age) constitute the HIV Incidence Risk Index for men who have sex with men (HIRI-MSM), a 7-item risk score developed by Smith et al. [24] to predict HIV seroconversion among GBM that is recommended by the Centers for Disease Control and Prevention to screen individuals who should be evaluated for PrEP use [25]. Scores < 10 and ≥ 10 were considered as “low risk” and “high risk”, respectively [24, 25].

Based on responses to items on gender identity and sexual orientation, participants were allocated in three groups: (1) key populations (sample 1): GBM, transgender and non-binary individuals recruited during October 2019 via Grindr; (2) key populations (sample 2): GBM, transgender and non-binary individuals recruited during February and March 2020 via Hornet; (3) other populations: heterosexual cisgender men and cisgender women.

### Statistical analysis

Socio-demographic characteristics of key populations (samples 1 and 2) and other populations were described. Procedures to assess differential item functioning followed a previously described protocol [26]. We first tested the original factor structure of the Perceived Risk of HIV Scale for the three groups separately using confirmatory factor analysis (CFA), using the weighted least squares estimator with a diagonal weight matrix, robust standard errors, and a mean- and variance-adjusted chi-square statistic with delta parameterization [27]. To assess model fit, the chi-square test, Tucker-Lewis Index (TLI) [28], Comparative Fit Index (CFI) [29] and Root Mean Square Error of Approximation (RMSEA) [30] were used. Since the chi-square test is highly sensitive to sample size, it can lead to the rejection of well-fitting models [31]. Therefore, the TLI, CFI and RMSEA fit indices were emphasized. Good fitting models were indicated by a TLI and CFI ≥ 0.95 and RMSEA ≤ 0.06 [32]. Once the factor structure was established for each group separately, two CFA models were fit combining participants from (1) key populations from sample 1 and other populations and (2) key populations from sample 2 and other populations. Modification indices were used to identify pairs of items for which model fit would improve if error estimates were freed to covary and for which there was theoretical justification [27]. We used theta parameterization after inclusion of error terms for correlated items [33].

To determine whether the Perceived Risk of HIV Scale items exhibited DIF for participants from key populations versus other populations, multiple indicator multiple cause (MIMIC) models were used. MIMIC models assess the relationship between the factors and a set of covariates to understand measurement invariance and population heterogeneity. The base MIMIC model consists of the CFA factor model with an added direct effect of group on the latent factors. This serves to control for group differences on the latent factor. We followed standard procedures for MIMIC analyses [26] which consist of regressing each item separately on the grouping variable to assess potential DIF. The presence of substantive DIF could threaten the validity of group comparisons. DIF was confirmed by a statistically significant (*p* < 0.05) link of group with the item, controlling for differences in the overall level of the latent factor. Once all items with significant DIF were identified, the magnitude of DIF items collectively was evaluated by comparing the difference on the latent factor between groups in the baseline model and after controlling for DIF.

We estimated the scale’s internal consistency using Cronbach’s alpha (assumed acceptable if > 0.7) [34]. To assess concurrent validity, we used t-tests to evaluate if participant’s scores on the Perceived Risk of HIV Scale differed by age (18–24 vs. ≥ 25 years) and by prior HIV testing (yes vs. no) for key populations and other populations separately. We hypothesized that younger individuals (18–24 years vs. ≥ 25 years) and those who had previously tested for HIV (vs. not) would score higher on the Perceived Risk of HIV scale. Lastly, we used t-tests to evaluate if Perceived Risk of HIV scores were higher among key populations from sample 2 who reported engaging in high-risk sexual behaviors measured by condomless receptive anal sex (yes vs. no), number of male partners (≤ 5 vs. > 5) and the HIRI-MSM risk scale (low risk vs. high risk).

CFA and MIMIC analyses were carried out in Mplus version 8.4 [27]; all other analyses were performed in R version 4.0.1 [35].

## Results

### Step 1: Translation

The qualitative pretesting of the final translated version was performed with 80 GBM, most were aged ≥ 25 years (79%), had finished college or higher education (74%), and lived in the city of Rio de Janeiro (69%). Most participants (ranging from 89 to 100%) judged each item’s clarity with a score of 7 or higher; average clarity scores varied from 9.4 for item 1 to 9.8 for item 2, 7 and 8. Item 1 was slightly modified as a function of the suggestions made by participants. The post-pretesting version of the translated scale is given in Table 1.

**Table 1:** The 8 final items of the translated Perceived Risk of HIV Scale with item-specific response options given in parenthesis

##	Original item	Range	Final Brazilian Portuguese translation
i01	What is your gut feeling about how likely you are to get infected with HIV? (Extremely unlikely to Extremely likely)^a^	1–4	Na sua opinião, qual sua chance de pegar HIV? (Extremante improvável a Extremamente provável)
i02	I worry about getting infected with HIV. (None of the time to All of the time)^a^	1–4	Eu me preocupo se vou pegar HIV (Nunca a sempre)
i03	Picturing self getting HIV is something I find: (Very hard to do to Very easy to do)^b^	1–4	Me imaginar pegando HIV é algo que acho: (Muito difícil a Muito fácil)
i04	I am sure I will NOT get infected with HIV. (Strongly disagree to Strongly agree)^c,d^	1–6	Tenho certeza de que NÃO vou pegar HIV (Discordo fortemente a Concordo fortemente)
i05	I feel vulnerable to HIV infection. (Strongly disagree to Strongly agree)^a^	1–6	Me sinto vulnerável à infecção pelo HIV (Discordo fortemente a Concordo fortemente)
i06	There is a chance, no matter how small, I could get HIV. (Strongly disagree to Strongly agree)^c^	3–6	Existe uma chance, ainda que mínima, que eu pegue HIV (Discordo fortemente a Concordo fortemente)
i07	I think my chances of getting infected with HIV are: (Zero to Very large)^c^	1–6	Acho que meu risco de pegar HIV é: (Zero a Muito alta)
i08	Getting HIV is something I have: (Never thought about to Thought about often)^b^	1–4	Pegar HIV é algo em que: (Eu nunca pensei a Pensei frequentemente)

^a^Affective item

^b^Salience item

^c^Cognitive item

^d^Reversed coded

### Step 2: Validation

#### Sample characteristics

In October 2019, a total of 2311 individuals accessed the questionnaire and 1358 (59%) completed all items. Of these, 390 were randomized to complete the translated version of the Perceived Risk of HIV Scale (Fig. 1), including 235 from key populations (sample 1) and 155 from other populations. During February and March 2020, a total of 10,708 participants accessed the questionnaire, and 6559 completed it. Of these, 4107 self-reported HIV negative or unknown status and were not currently using PrEP. Socio-demographic characteristics for key populations (sample 1 and sample 2) and other populations are displayed in Table 2.

**Table 2:** Characteristics of study populations. Brazil, 2019–2020

	Key populations		Other populations
	Sample 1	Sample 2	
Total	235	4107	155
Age			
Mean (SD)	34.6 (10.1)	34.2 (10.4)	46.9 (16.1)
18–24 years	37 (15.7)	681 (16.6)	16 (10.3)
≥ 25 years	198 (84.3)	3426 (83.4)	139 (89.7)
Gender			
Cisgender men	209 (97.4)	4024 (98.0)	29 (18.7)
Cisgender women	0 (0)	0 (0)	126 (81.3)
Trans/non-binary	6 (2.6)	83 (2.0)	0 (0)
Sexual orientation			
Gay	186 (79.1)	3365 (81.9)	0 (0)
Lesbian	0 (0)	0 (0)	7 (4.5)
Bisexual	45 (19.1)	720 (17.5)	16 (10.3)
Heterosexual	4 (1.7)	22 (0.5)	132 (85.2)
Race			
White	135 (57.4)	2526 (63.1)	102 (68.0)
Black	27 (11.5)	394 (9.8)	12 (8.0)
Pardo (mixed)/native	73 (31.1)	1085 (27.1)	36 (24.0)
Income^a^			
Low	80 (34.0)	1184 (28.8)	43 (27.7)
Middle	78 (33.2)	1853 (45.1)	75 (48.4)
High	77 (32.8)	1070 (26.1)	37 (23.9)
Education (years)^b^			
≤ 12	75 (32.1)	1278 (31.7)	59 (38.1)
> 12 (college or higher)	159 (67.9)	2754 (68.4)	96 (61.9)
Region			
North, Northeast and Central-west	74 (31.5)	319 (7.8)	27 (17.4)
Southeast/South	161 (68.5)	3788 (92.2)	128 (82.6)
Current partner			
No	171 (72.8)	2818 (71.3)	52 (33.5)
Yes	64 (27.2)	1135 (28.7)	103 (66.5)
Last HIV test			
Past 3 months	83 (35.3)	1120 (27.3)	10 (6.5)
Past 6 months	34 (14.5)	794 (19.3)	14 (9.0)
Past 12 months	32 (13.6)	739 (18.0)	23 (14.8)
More than 12 months	56 (23.8)	948 (23.1)	64 (41.3)
Never	30 (12.8)	506 (12.3)	44 (28.4)
Score			
Mean (SD)	25.8 (5.2)	25.7 (4.9)	20.5 (5.5)

^a^We considered the number of minimum wages in the family monthly income: low ≤ 2, middle > 2–6, high > 6 (monthly minimum wage in 2019 was 998 BRL = US$ 248, currency from January 2020)

^b^≤ 12 years is equivalent to complete Secondary Education or less, > 12 is equivalent to complete College education or higher

*Key populations (Sample 1)* In total, 235 GBM and trans/non-binary participants completed the translated version of the Perceived Risk of HIV Scale. Median age was 33 years (IQR: 27–41), the majority were cisgender men (97%), 186 (79%) were gay, 135 (57%) were White, 80 (34%) reported low-income, 159 (68%) reported college education or higher. The Perceived Risk of HIV Scale mean score was 25.8 (SD 5.2).

*Key populations (Sample 2)* In total, 4107 GBM and trans/non-binary participants completed the translated version of the Perceived Risk of HIV Scale. Median age was 32 years (IQR: 26–40), the majority were cisgender men (98%), 3365 (82%) were gay, 2526 (63%) were White, 1184 (29%) reported low-income, 2754 (68%) reported college education or higher. According to the HIRI-MSM scale, more than a half of participants could be classified as engaging in high-risk behavior (2276, 55%); 1527 (37%) reported condomless receptive anal sex and 1650 (40%) reported more than five male sexual partners in the past 6 months. The Perceived Risk of HIV Scale mean score was 25.7 (SD 4.9).

*Other populations* In total, 155 participants completed the translated version of The Perceived Risk of HIV Scale. Median age was 48 years (interquartile range [IQR]: 35–59), the majority were cisgender women (81%), 132 (85%) were heterosexual, 102 (68%) were White, 43 (28%) reported low-income, 96 (62%) reported college education or higher. The Perceived Risk of HIV Scale mean score was 20.5 (SD 5.5).

Comparing the groups (Table 2), we note that participants from key populations were younger, almost all cisgender men (versus cisgender women), gay or bisexual (versus heterosexual), reported higher education though were more likely to be low income, were less likely to have current partner, more likely to have ever tested for HIV, and scored higher in the Perceived Risk of HIV scale than other populations.

### Confirmatory factor analysis and differential item functioning

Factor loadings of the one-factor structure CFA models for key populations sample 1 and 2 and other populations are provided in Table 3. Model’s fit for the groups were (1) key populations (sample 1): chi-square (20) = 94.7, *p* ≤ 0.001, CFI = 0.95, TLI = 0.92, RMSEA = 0.13 (90% CI 0.10–0.15); (2) key populations (sample 1): chi-square (20) = 1137.1, *p* ≤ 0.001, CFI = 0.94, TLI = 0.92, RMSEA = 0.12 (90% CI 0.11–0.12); (3) other populations: chi-square (20) = 93.8, *p* ≤ 0.001, CFI = 0.92, TLI = 0.89, RMSEA = 0.15 (90% CI 0.12–0.19). Of note is that the loadings for item 2 were within the expected range for other populations (0.59) but small for key populations (sample 1: 0.20 and sample 2: 0.03).

**Table 3:** Factor loadings of the one-factor CFA models for the Perceived Risk of HIV Scale for key populations sample 1 and 2 and other populations

Items	Key populations		Other populations
	Sample 1	Sample 2	
i01. What is your gut feeling about how likely you are to get infected with HIV?	**0.741**	**0.766**	**0.795**
i02. I worry about getting infected with HIV	0.201	0.033	**0.590**
i03. Picturing self getting HIV is something I find	**0.752**	**0.646**	**0.560**
i04. I am sure I will NOT get infected with HIV	**0.636**	**0.635**	**0.593**
i05. I feel vulnerable to HIV infection	**0.628**	**0.632**	**0.637**
i06. There is a chance, no matter how small, I could get HIV	**0.400**	**0.538**	**0.555**
i07. I think my chances of getting infected with HIV are:	**0.918**	**0.868**	**0.853**
i08. Getting HIV is something I have:	**0.375**	**0.378**	**0.664**
Fit indices			
Chi-square test of model fit	94.7	1137.1	93.8
CFI	0.95	0.94	0.92
TLI	0.92	0.92	0.89
RMSEA (90% confidence interval)	0.13 (0.10–0.15)	0.12 (0.11–0.12)	0.15 (0.12–0.19)

Bold indicate *p* value ≤ 0.001

The first single-factor model was fit combining sample 1 key populations and other populations, including a direct effect of group on the latent factor. Results indicate a marginally acceptable fit [chi-square (27) = 241.7, *p* ≤ 0.001, CFI = 0.91, TLI = 0.89, RMSEA = 0.14 (90% CI 0.13–0.16)]. Inspection of modification indices suggested that error terms belonging to items 2 and 8 might be correlated. The re-specified model including the correlation of these items had a moderate acceptable fit [chi-square (26) = 194.6, *p* ≤ 0.001, CFI = 0.93, TLI = 0.91, RMSEA = 0.13 (90% CI 0.11–0.15)].

Prior to accounting for possible DIF, key populations participants (sample 1) had 1.29 SD higher latent factor levels than other populations (see Table 4, *p* ≤ 0.001). DIF analyses indicated that items 2, 4 and 8 had statistically significant DIF (*p* ≤ 0.001), with items 2 and 8 being endorsed at higher levels by key populations and item 4 by other populations. As shown in Table 4, after correcting for DIF, key populations had 1.19 SD higher latent factor levels compared to other populations (i.e., the effect of DIF on overall scores was of 0.10 standard deviations). The final model accounting for DIF had a good fit [chi-square (23) = 62.5, *p* ≤ 0.001, CFI = 0.98, TLI = 0.98, RMSEA = 0.07 (90% CI 0.05–0.09)].

**Table 4:** Factor loadings for the Perceived Risk of HIV Scale in key populations (sample 1) and other populations and influence of group on the overall estimates of latent factor score

Items	Base model^a^		DIF corrected model^b^	
	Factor loading	95%CI	Factor loading	95%CI
i01. What is your gut feeling about how likely you are to get infected with HIV?	0.808	0.76, 0.85	0.806	0.77, 0.84
i02. I worry about getting infected with HIV	0.406	0.30, 0.51	0.284	0.20, 0.37
i03. Picturing self getting HIV is something I find	0.710	0.65, 0.77	0.706	0.66, 0.76
i04. I am sure I will NOT get infected with HIV	0.642	0.58, 0.70	0.685	0.62, 0.75
i05. I feel vulnerable to HIV infection	0.678	0.62, 0.74	0.673	0.62, 0.72
i06. There is a chance, no matter how small, I could get HIV	0.512	0.43, 0.59	0.508	0.44, 0.58
i07. I think my chances of getting infected with HIV are	0.914	0.88, 0.94	0.913	0.89, 0.94
i08. Getting HIV is something I have	0.511	0.42, 0.60	0.431	0.35, 0.51
Structural effect				
Group on latent factor^c^	**1.287**	**0.96, 1.61**	**1.192**	**0.87, 1.51**
Direct effect on item attributable to group^c^				
i02			**1.041**	**0.78, 1.30**
i04			**− 0.504**	**− 0.76, − 0.25**
i08			**0.616**	**0.37, 0.86**

Bold indicate *p* value ≤ 0.001

*DIF* differential item functioning

^a^Not corrected for DIF

^b^Correct for DIF for items 2, 4 and 8

^c^Other populations used as reference

The second single-factor model was fit combining the sample 2 key populations and other populations, including a direct effect of group on the latent factor. Results indicate a marginally acceptable fit [chi-square (27) = 1722.7, *p* ≤ 0.001, CFI = 0.92, TLI = 0.89, RMSEA = 0.12 (90% CI 0.12–0.13)]. Similar to results above, inspection of modification indices suggested that error terms belonging to items 2 and 8 should be correlated. The re-specified model including the effect of these items had a marginally acceptable fit [chi-square (26) = 1323.8, *p* ≤ 0.001, CFI = 0.94, TLI = 0.92, RMSEA = 0.11 (90% CI 0.10–0.11)].

Prior to accounting for possible DIF, key populations participants (sample 2) had 1.12 SD higher latent factor levels than other populations (see Table 5, *p* ≤ 0.001). DIF analyses also indicated that items 2, 4 and 8 were statistically significant (*p* ≤ 0.001). As shown in Table 5, after correcting for DIF, key populations had 1.14 SD higher latent factor levels compared to other populations (i.e., the effect of DIF on overall scores was of 0.02 standard deviations). The final model accounting for DIF had a good fit [chi-square (23) = 729.9, p ≤ 0.001, CFI = 0.97, TLI = 0.95, RMSEA = 0.09 (90% CI: 0.08–0.09)].

**Table 5:** Factor loadings for the Perceived Risk of HIV Scale in key populations (sample 2) and other populations and influence of group on the overall estimates of latent factor score

Items	Base model^a^		DIF corrected model^b^	
	Factor loading	95%CI	Factor loading	95%CI
i01. What is your gut feeling about how likely you are to get infected with HIV?	0.773	0.76, 0.79	0.773	0.76, 0.79
i02. I worry about getting infected with HIV	0.054	0.01, 0.10	0.023	− 0.02, 0.07
i03. Picturing self getting HIV is something I find	0.649	0.63, 0.67	0.649	0.63, 0.67
i04. I am sure I will NOT get infected with HIV	0.636	0.62, 0.65	0.644	0.62, 0.66
i05. I feel vulnerable to HIV infection	0.639	0.62, 0.66	0.639	0.62, 0.66
i06. There is a chance, no matter how small, I could get HIV	0.545	0.52, 0.57	0.545	0.52, 0.57
i07. I think my chances of getting infected with HIV are:	0.871	0.86, 0.88	0.871	0.86, 0.88
i08. Getting HIV is something I have	0.392	0.35, 0.42	0.383	0.35, 0.41
Structural effect				
Group on latent factor^c^	**1.119**	**0.91, 1.33**	**1.143**	**0.93, 1.36**
Direct effect on item attributable to group^c^				
i02			**1.675**	**1.50, 1.86**
i04			**− 0.552**	**− 0.73, − 0.38**
i08			**0.550**	**0.38, 0.72**

Bold indicate *p* value ≤ 0.001

*DIF* differential item functioning

^a^Not corrected for DIF

^b^Correct for DIF for items 2, 4 and 8

^3^Other populations used as reference

Internal consistency reliability for each group were: key populations sample 1 (*α* = 0.76; 95% CI 0.71–0.80), key populations sample 2 (*α* = 0.75; 95% CI 0.74–0.76), and other populations (*α* = 0.78; 95% CI 0.73–0.83).

Construct validity results are shown in Table 6. We observed that participants who ever tested for HIV scored higher among key populations from sample 2 and other populations (*p* < 0.001, both samples). Perceived Risk of HIV Scale scores for key populations of sample 2 were significantly higher among those engaging in high-risk sexual behavior irrespective of how high-risk behavior was measure (*p* < 0.001). Younger individuals scored lower than older individuals among key populations (sample 1: *p* = 0.09; sample 2: *p* = 0.005).

**Table 6:** Perceived Risk of HIV Scale scores according to age, HIV testing and HIV risk behavior. Brazil, 2020

	Other populations			Key populations, sample 1			Key populations, sample 2		
	N = 155	Score mean (SD)	*p* value	N = 235	Score mean (SD)	*p* value	N = 4107	Score mean (SD)	*p* value
Age (years)			0.39			0.09			0.005
18–24	16	21.6 (6.2)		37	24.4 (5.3)		681	25.2 (5.0)	
≥ 25	139	20.4 (5.4)		198	26.0 (5.1)		3426	25.8 (4.9)	
HIV testing			< .001			0.26			< .001
Never	44	17.9 (5.0)		30	24.8 (5.9)		506	24.8 (5.0)	
At least once lifetime	111	21.5 (5.3)		205	25.9 (5.1)		3601	25.8 (4.9)	
Condomless receptive anal sex									< .001
No	*	*	*	*	*	*	2580	25.0 (4.9)	
Yes	*	*	*	*	*	*	1527	27.0 (4.7)	
Number of male partners									< .001
≤ 5	*	*	*	*	*	*	2457	24.8 (4.8)	
> 5	*	*	*	*	*	*	1650	27.0 (4.7)	
HIRI-MSM scale									< .001
Low	*	*	*	*	*	*	1831	24.4 (4.8)	
High	*	*	*	*	*	*	2276	26.7 (4.8)	

*Data not available

## Discussion

In this study, we successfully performed a cross-cultural adaptation of the Brazilian Portuguese version of the Perceived Risk of HIV Scale. CFA showed that the single-factor structure of the scale had sufficiently good fit among all groups considered. Significant DIF was found for 3 items, and key populations had substantially higher latent factor levels compared to other populations. Our results indicate that this scale can be used among key populations in Brazil, such as GBM and transgender/non-binary individuals as well as other populations.

An important finding was that, for the two comparisons between key populations and other populations, three items of the Perceived Risk of HIV Scale (item 2: “I worry about getting infected with HIV”, item 4: “I am sure I will not get infected with HIV”, and item 8: “Getting HIV is something I have”) exhibited statistically significant DIF. However, the effect on overall scores of the Perceived Risk of HIV scale was negligible (0.10 SD and 0.02 SD for the models considering other populations and key populations sample 1 and 2, respectively). Key populations had higher Perceived Risk of HIV scale scores on items 2 and 8 and lower scores on item 4. These results suggest that scores from key populations and other populations can be validly compared and assumed to be measuring risk perception using the same metric. DIF would also negligibly influence overall scores.

The HIV epidemic in Brazil is highly concentrated on key populations including GBM and TGW, with HIV prevalence being 10–30 times higher among these populations [2, 4, 5]. Since the onset of the epidemic, HIV has been portrayed as a disease of gay men. Studies suggests that cultural constructions of sexuality influence an individual’s perception of HIV risk and vulnerability [36]. In an analysis of individual- and partner-level factors associated with partner-specific perceptions of risk among GBM and TGW who had recently acquired HIV infection, both gay or transgender partners were more likely to be considered a source of infection than heterosexual partners [37]. These findings suggest a latent cultural logic that identifies GBM or TGW as “carriers of HIV disease” [36] independent of their actual objective risk of acquiring or transmitting HIV. We hypothesize that this association of HIV infection with GBM coupled with Brazil’s heterosexist and transphobic society might explain key populations’ much higher endorsement of item 2, “I worry about getting infected with HIV”. Item 2 was endorsed by more than half of the participants from key populations (50.6% sample 1 and 56.5% sample 2 responding that they “worry about getting HIV all of the time”) whereas more than half of participants from other populations reported “worrying about getting HIV none of the time (24.5%) and rarely (29.0%)”. This line of reasoning could also explain why the factor loading for item 2 was small among key populations (sample 1: 0.20 and sample 2: 0.03 compared to 0.54 for other populations, Table 3). A similar pattern may be observed for item 8 (“Getting HIV is something I have”) which more than 80% of the participants from key populations (85.1% of sample 1 and 82.5% of sample 2) responding that getting HIV is something they have “thought about often or sometimes” whereas almost half of participants from other populations answered “rarely” (24.6%) and “never” (23.9%) explaining the relatively low factor loadings for key populations (sample 1 and sample 2: 0.38 compared to 0.66 for other populations). Indeed, items 2 and 8 were found to be correlated in the CFA analysis. Taken together, these findings suggest that HIV is something key populations constantly think and worry about independently of their objective assessments of risk or of how salient HIV is to them. Furthermore, these results highlight the importance of measuring the different dimensions of risk perception as highlighted not only in regards to HIV [18] but also more broadly [38]. Finally, though the low factor loadings for items 2 and 8 could suggest that they be removed from the scale when assessing perceived HIV risk among key populations (thus consequently creating a different measure for key populations), we argue for its maintenance because it appropriately loaded in other populations, and to keep with a single uniform instrument that may be applied to all populations.

Confirming our hypotheses, among key populations from sample 2, for whom we had information on sexual behavior, participants who reported high risk sexual behavior (condomless receptive anal sex, > 5 sexual partners and ≥ 10 points in the HIRI-MSM risk score) scored higher on the Perceived Risk of HIV Scale than those who reported low risk behavior. These results suggest that perceived HIV risk, as measured by the proposed translated version of the scale, correlates with sexual behavior as supposed in theoretical models [14].

We found that participants who ever tested for HIV scored higher on the Perceived Risk of HIV scale than those who never tested for key populations and other populations. This positive correlation between scores in the Perceived Risk of HIV scale and ever testing for HIV was also found during the validation of the present scale to European Portuguese, which was conducted in the general population and with HIV-uninfected partners from serodifferent couples [39], and this association is corroborated by prior systematic review findings [40]. We also found that among key populations, younger participants scored lower on the Perceived Risk of HIV scale. Younger GBM aged 18–24 years reported lower perceived HIV risk in comparison to older GBM in a prior analysis in Brazil [8, 41]. The possible misperception of HIV risk among the youngest could help explain the recent rise in HIV incidence among GBM aged 16–24 years in Brazil [2]. Another explanation for this finding is that younger individuals may be more optimistic of HIV treatment and prevention strategies and less fearful of HIV infection than older peers [42–44]. Although these results are of importance for the concurrent validity of Brazilian Portuguese version of The Perceived Risk of HIV Scale, these associations should be confirmed by larger studies in particular when thinking of the application of the scale in other populations for which our sample was small.

There are several study limitations to highlight. There was a difference in sample size between the groups. However, this difference may not have influenced the results as the initial confirmatory factor analysis yielded the similar model structures. Due to the cross-sectional design of the study, test–retest reliability and sensitivity to change of the translated HIV Perceived Risk scale could not be assessed. All collected data were self-reported by participants and may be subject to bias (social desirability bias). Also, participants were recruited from a convenience online sample and may not reflect the entire Brazilian population, although cellphones and internet connection are widely available in all socioeconomic strata of the Brazilian population [45, 46]. Finally, a limitation of the MIMIC model we used is that it assessed uniform but not non-uniform DIF.

## Conclusions

The HIV Perceived Risk Scale was effectively cross-culturally adapted to Brazilian Portuguese and validated. This instrument has shown to be practical, easy to administer, and could be useful in future studies to better measure perceived HIV risk and its association with engagement in high-risk sexual behaviors among a plurality of populations from Brazil.

## Supplementary Information

**Additional file 1**. Escala de Percepção de Risco do HIV em Português do Brasil.

## Data Availability

The datasets analyzed during the current study are available from the corresponding author on reasonable request.

## Abbreviations

AIDS: Acquired immunodeficiency syndrome
CFA: Confirmatory factor analysis
CFI: Comparative Fit Index
CI: Confidence interval
DIF: Differential item functioning
GBM: Gay, bisexual and other men who have sex with men
HIRI-MSM: HIV Incidence Risk Index for men who have sex with men
HIV: Human immunodeficiency virus
IQR: Interquartile range
MIMIC: Multiple indicator multiple cause
PrEP: Pre-exposure prophylaxis
RMSEA: Root Mean Square Error of Approximation
TLI: Tucker-Lewis Index
TGW: Transgender women who have sex with men
