# Supplementary material for: Cross-cultural adaptation of the Perceived Risk of HIV Scale in Brazilian Portuguese
Source: Health Qual Life Outcomes. 2021 Apr 9;19:117. doi: 10.1186/s12955-021-01760-6 (PMC8033701; doi:10.1186/s12955-021-01760-6)
Supplement: Supplementary file 1 — Additional file 1. Escala de Percepção de Risco do HIV em Português do Brasil. [file 12955_2021_1760_MOESM1_ESM.pdf]

Escala de Percepção de Risco do HIV em Português do Brasil

1. Na sua opinião, qual sua chance de pegar HIV?

- extremamente improvável (1)
- muito improvável (2)
- um pouco provável (3)
- muito provável (4)
- extremamente provável (4)

2. Eu me preocupo se vou pegar HIV

- Nunca (1)
- Raramente (2)
- às vezes (3)
- boa parte do tempo (4)
- quase sempre (4)
- sempre (4)

3. Me imaginar pegando HIV é algo que acho

- muito difícil (1)
- difícil (2)
- fácil (3)
- muito fácil (4)

4. Tenho certeza de que NÃO vou pegar HIV

- discordo fortemente (6)
- discordo (5)
- discordo em parte (4)
- concordo em parte (3)
- concordo (2)
- concordo fortemente (1)

5. Me sinto vulnerável à infecção pelo HIV

- discordo fortemente (1)
- discordo (2)
- discordo em parte (2)
- concordo em parte (4)
- concordo (5)
- concordo fortemente (6)

6. **Existe uma chance, ainda que mínima, que eu pegue HIV**
- discordo fortemente (3)
- discordo (3)
- discordo em parte (3)
- concordo em parte (4)
- concordo (5)
- concordo fortemente (6)

7. **Acho que meu risco de pegar HIV é:**
- zero (1)
- quase zero (2)
- baixo (3)
- moderado (4)
- alto (5)
- muito alto (6)

8. **Pegar HIV é algo em que**
- eu nunca pensei (1)
- raramente pensei (2)
- pensei algumas vezes (3)
- pensei frequentemente (4)

Traduzido e validado de:

Napper et al. Development of the perceived risk of HIV scale. AIDS Behav 2012;16(4):1075-83. doi: 10.1007/s10461-011-0003-2.
